# A cross-sectional study on the association between secondhand smoke exposure and suicide among adolescents in multicultural families: The mediating role of perceived stress

**DOI:** 10.18332/tid/209551

**Published:** 2025-10-21

**Authors:** Wenbin Du, Yu Luo, Yunyun Wu, Yuxi Wang

**Affiliations:** 1 Research Institute of Social Development, Southwestern University of Finance and Economics, Chengdu, China; 2 Department of International Relations, Yonsei University, Wonju, Republic of Korea

**Keywords:** SHS exposure, South Korea, suicidality, multicultural family adolescents

## Abstract

**INTRODUCTION:**

Adolescent suicidality poses a serious barrier to healthy growth and development. This study examines the association between secondhand smoke (SHS) exposure and suicidality among adolescents from multicultural families in South Korea, using a secondary analysis of the Korea Youth Risk Behavior Webbased Survey (KYRBS).

**METHODS:**

This cross-sectional study used pooled secondary data from the 2021 and 2024 waves of the KYRBS, a nationally representative survey of adolescents in South Korea, collected through self-administered questionnaires. This is a secondary dataset analysis of the KYRBS using logit regression models to assess the associations between the duration and setting-specific frequency of SHS exposure and suicidality among adolescents. The mediating role of perceived stress was examined using the Karlson-Holm-Breen (KHB) method.

**RESULTS:**

SHS exposure is significantly associated with increased suicidality likelihood among adolescents, with particularly strong associations observed in those from multicultural families. Among multicultural adolescents, each additional total day of SHS exposure is significantly associated with the likelihood of suicidal ideation (AOR=1.117; 95% CI: 1.084–1.151), suicide planning (AOR=1.095; 95% CI: 1.052–1.139), and suicide attempts (AOR=1.117; 95% CI: 1.069–1.168). SHS exposure showed a stronger association with suicidality in multicultural families versus non-multicultural families. A higher frequency of SHS exposure across multiple settings is significantly associated with elevated odds of suicidal ideation (AOR=1.422; 95% CI: 1.247–1.621), suicide planning (AOR=1.395; 95% CI: 1.153–1.689), and suicide attempts (AOR=1.524; 95% CI: 1.222–1.902). Further analysis reveals that perceived stress partially mediates the association between SHS exposure and suicidality among multicultural adolescents. Perceived stress indirectly mediated 23.19% of the effect of SHS exposure on suicide attempts. It also mediated 30.67% and 34.18% of the effects on suicidal ideation and planning, respectively.

**CONCLUSIONS:**

SHS exposure was associated with a higher likelihood of suicidality among adolescents, with this association observed in greater magnitude in adolescents from multicultural families. Moreover, perceived stress partially mediates the association between SHS exposure and suicide attempts among multicultural adolescents.

## INTRODUCTION

Suicide has emerged as the primary cause of mortality among adolescents in South Korea, presenting a significant public health challenge^1^. In South Korea, the rate of death due to suicide is significantly higher compared with that in other developed countries^2^. According to the ‘2020 Suicide Prevention White Paper’ data, South Korea’s teenage suicide rate increased from 4.2–4.9 per 100000 people in 2017 to 5.8 in 2018, making South Korean teenagers an age group with the highest rate of increase (22.1%). It is also 1.7 times higher than the average rate of suicide among teenagers in Organization for Economic Cooperation and Development (OECD) countries^3^. This issue is particularly pronounced among adolescents from multicultural families, who constitute a growing segment of the South Korean youth population due to the rising number of international marriages^4^. Multicultural families are defined as those where at least one parent is foreign-born^5^. In 2018, the adolescent population from multicultural families in South Korea numbered approximately 1222000, representing 2.2% of the total adolescent population, a threefold increase from 2012^6^. This escalating trend underscores the pressing need to address the mental health concerns of children from multicultural families. Research indicates that adolescents from multicultural backgrounds are at a heightened risk of mental health issues, with a study based on the 2015 Korea Youth Risk Behavior Web-based Survey revealing a significantly higher prevalence of suicidal ideation (15.8%) compared to their non-multicultural counterparts (11.3%)^7^. During adolescence, school difficulties, relationship problems with peers, and social maladjustment may lead to suicidal ideation and suicidal behavior^8^. Challenges such as language barriers, cultural adjustment difficulties, and social discrimination contribute to the elevated risk of suicidal ideation and behaviors among adolescents from multicultural families^9^. The implications of suicide among these adolescents extend beyond individual and familial repercussions to impact social cohesion and sustainable development in South Korean society^10^. As the number of multicultural families continues to rise, the educational, social, and psychological challenges faced by their children become increasingly salient^11^. Failure to effectively address the mental health needs of this population may exacerbate social marginalization, perpetuate educational disparities, and erode social cohesion^12^. Therefore, comprehensive research into the suicide risk factors and mental health vulnerabilities specific to adolescents from multicultural families, along with the development of culturally sensitive prevention and intervention strategies, are critical imperatives in the realms of public health and social policy.

When examining mental health issues among adolescents from multicultural families, smoking emerges as a significant contributing factor^13^. Previous epidemiological studies have revealed a higher prevalence of suicidal behavior among current smokers, indicating a potential link between smoking and suicide^14^. Specifically, research has identified a notable association between adolescent smoking and suicidal ideation and actions^15^. For instance, a multinational study demonstrated a significantly elevated likelihood of suicide attempts among adolescent smokers compared to non-smokers, with a greater magnitude of association observed in females^16^. Furthermore, the impact of secondhand smoke (SHS) exposure as a form of passive smoking has garnered attention from researchers. While research specifically on the relationship between SHS exposure and suicide in adolescents from multicultural families is lacking, existing studies have established a strong connection between SHS exposure and mental health issues in adolescents^17^. For example, an analysis of data from the Korean Youth Health Behavior Survey revealed a positive association between SHS exposure and stress and depression in adolescents^18^. Moreover, global research has supported this association, highlighting a dose-response relationship between SHS exposure and depressive symptoms in adolescents, with higher exposure frequencies increasing the risk of depressive symptoms^19^. Although investigations into the link between SHS exposure and suicide among adolescents from multicultural families are limited, the existing literature underscores the close relationship between SHS exposure and mental health problems in this population. Therefore, further research is warranted to explore the potential association between SHS exposure and suicide among adolescents from multicultural families, aiming to inform the development of targeted intervention strategies based on scientific evidence.

Recent studies have increasingly acknowledged perceived stress as a crucial psychosocial factor linking smoking exposure to negative mental health outcomes in adolescents. Multiple investigations have shown that increased perceived stress serves as a mediator between smoking habits and depressive symptoms in young individuals, underscoring its pivotal role in shaping psychological susceptibility^20,21^. Particularly among multicultural adolescents, who often confront distinct sociocultural obstacles like acculturative stress and discrimination, perceived stress has been recognized as a potential risk element that amplifies suicidal tendencies^12^. Nevertheless, there is a noticeable research gap in explicitly exploring how perceived stress mediates the association between SHS exposure and suicidality in this susceptible group, emphasizing the necessity for targeted inquiry.

The escalating prevalence of suicide among adolescents from multicultural backgrounds presents a pressing social and public health concern necessitating immediate attention. These individuals encounter heightened sociocultural adaptation challenges and mental health vulnerabilities compared to their peers in the general adolescent population^12^. Notably, smoking behavior and consequent exposure to SHS may significantly impact their psychological well-being^20^. Thus, this research investigates the association between SHS exposure and suicidal tendencies in adolescents from multicultural families, utilizing data from the Korea Youth Risk Behavior Web-based Survey (KYRBS). By specifically examining this demographic group, the study aims to explain potential pathways of mental health risks – particularly the mediating role of perceived stress – and provide empirical evidence that may inform future research and intervention strategies to support the overall welfare of multicultural adolescents.

## METHODS

### Data sources and sample selection

The Korea Youth Risk Behavior Web-based Survey (KYRBS), conducted annually since 2005 by the Korea Disease Control and Prevention Agency (KDCA), is a nationally representative dataset for studying adolescent risk behaviors in South Korea. The KYRBS employs an annual cross-sectional design and collects self-reported data through standardized, web-based questionnaires administered in school settings. This study is a secondary analysis using data from the 17th (2021) and 20th (2024) waves of the survey. We selected the 2021 and 2024 waves of the KYRBS for analysis based on both the availability and consistency of key variables. Specifically, 2021 was the first year to include comprehensive measures of tobacco product use, including combustible cigarettes, nicotine-containing e-liquids, and heated tobacco products. Additionally, information on SHS exposure in schools was only available in 2021 and 2024. These criteria ensured that the main independent and exposure variables were measured consistently across both years.

The survey employed stratified cluster sampling to select 800 schools – 400 middle schools and 400 high schools – targeting students from Grade 1 of middle school to Grade 3 of high school across South Korea. In 2021, 59426 students were sampled and 54848 participated (response rate: 92.9%). In 2024, 57580 were sampled and 54653 responded (response rate: 94.9%). The KYRBS data are publicly available for download from the official website (https://www.kdca.go.kr).

**Figure 1:**
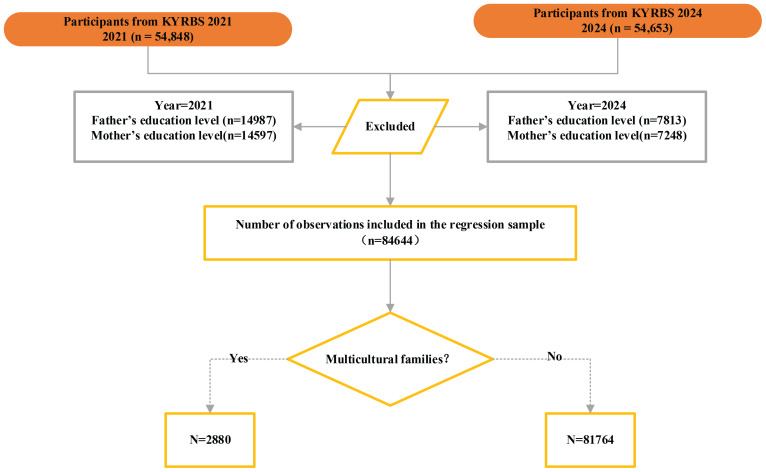
Flowchart of participants selection, South Korea (2021–2024)

This study focused on adolescents from multicultural families, defined as having parents from different countries or at least one parent born outside South Korea. The 2021 and 2024 KYRBS datasets were combined to create a pooled cross-sectional dataset to examine the association between SHS exposure and suicidality among Korean adolescents. After excluding 24857 cases with missing data, the final analytical sample included 84644 observations – 2880 from multicultural families and 81764 from non-multicultural families. The sample selection process is illustrated in Figure 1.

### Measurement instruments

*Outcome variable*

The dependent variable is suicidality among South Korean adolescents, assessed across three dimensions: suicidal ideation, suicide planning, and suicide attempts, based on the following survey items: ‘During the past 12 months, have you seriously thought about suicide?’, ‘During the past 12 months, have you made any specific plans to commit suicide?’, and ‘During the past 12 months, have you attempted suicide?’. Responses of ‘yes’ were coded as 1, and ‘no’ as 0. Specifically, suicidal ideation reflects initial cognitive engagement with suicidal thoughts; suicide planning indicates the development of specific strategies; and suicide attempts represent the most direct and severe behavioral expression of suicidality.

*Predictive variables*

The independent variable is SHS exposure. It is measured using two dimensions: the number of exposure days and the locations of exposure. Specifically, this variable is derived from responses to the following three survey items: ‘During the past 7 days, on how many days did you inhale smoke from someone else’s cigarette at home?’, ‘During the past 7 days, on how many days did you inhale smoke from someone else’s cigarette inside your school (e.g. classrooms, bathrooms, hallways)?’, and ‘During the past 7 days, on how many days did you inhale SHS in indoor places other than home or school (e.g. shops, restaurants, malls, PC rooms, karaoke bars)?’. The original responses were coded on a scale from 1 to 8, where 1 represented zero days of exposure and 8 represented seven days of exposure. To reflect the actual number of exposure days, we subtracted 1 from each response, thereby converting the scale to range from 0 to 7 days. We created three separate variables indicating the number of exposure days at home, at school, and in other indoor settings (excluding home and school).

*Total days of SHS exposure per week*

A cumulative SHS exposure variable was calculated by summing exposure days across home, school, and other indoor environments. This variable, treated as continuous, ranges from 0 to 21 days. Higher scores indicate greater cumulative SHS exposure across multiple indoor settings.

*SHS exposure locations and weekly SHS exposure location diversity*

Dummy binary variables were constructed to indicate the presence of SHS exposure in each setting (home, school, and other indoor environments). Exposure in each setting was coded as 1 if exposure occurred on one or more days, and 0 otherwise. A summary index of SHS exposure diversity was calculated by summing the three binary variables. The resulting variable ranged from 0 to 3, representing exposure at 0, 1, 2, or 3 distinct locations. Higher values represent greater diversity in SHS exposure environments.

By measuring both the duration of exposure (total days of SHS exposure per week) and the diversity of exposure settings (SHS exposure location diversity), these indicators capture the temporal intensity and contextual complexity of SHS exposure among adolescents.

*Controlled variables*

This study incorporated a range of covariates potentially associated with adolescents’ suicidality, structuring the control variables across three dimensions: personal characteristics, family background, and school environment. At the personal level, covariates included gender, academic performance, smoking behavior, grade level, and living arrangement. For academic performance, reverse coding was applied; on a scale of 1–5, a higher score indicates better academic achievement. Smoking behavior was treated as a binary dummy variable. Grade level ranged from the first year of middle school to the third year of high school. Living arrangement was categorized into five groups: living with family members, staying with relatives, renting independently (including with friends), residing in a dormitory, and living in a child welfare institution (e.g. orphanage, social welfare facility, or childcare center). At the family background level, covariates included family socio-economic status (SES), father’s education level, and mother’s education level. Family SES was measured on a subjective 1–5 scale, with higher scores indicating higher SES. At the school level, variables included school type and educational stage. School type was categorized into three groups: Girls’ schools, Boys’ schools, and Co-educational schools. Educational stage was classified as middle school or high school. These variables were included as potential confounders in the regression models to control for their possible effects on the associations between SHS exposure and adolescent suicidality.

Academic achievement (rated 1–5, indicating an ordinal scale from poor to excellent), grade level (ranging from 1 to 6, covering the first year of middle school to the third year of high school), and family economic status (rated 1–5, representing subjective socio-economic perception) were treated as ordinal variables. All other variables were treated as categorical variables.

*Mediating variable*

Perceived stress refers to an individual’s subjective assessment of stress. Perceived stress was operationalized based on responses to the question: ‘How much stress do you usually feel?. The five response categories were reverse-coded to reflect increasing levels of perceived stress. The original response options were: ‘I feel it deeply’, ‘I feel it quite a bit’, ‘I feel it a little’, ‘I don’t feel it very much’, and ‘I don’t feel it at all’. After reverse coding, these responses were transformed into a categorical variable ranging from 1 to 5. Higher values indicated greater levels of perceived stress.

### Statistical analysis

Firstly, we conducted descriptive analyses to provide an overview of all variables. Meanwhile, we calculated the annual percentages of suicidal tendencies across different types of families. Additionally, we generated stacked charts to analyze the relationships between various levels of SHS exposure and suicidal tendencies. Secondly, since all three variables measuring suicidal tendencies are dummy variables, using the ordinary least squares (OLS) model would lead to heteroscedasticity. Therefore, this study employed the logit model for regression analysis to examine the relationships between the duration of SHS exposure, exposure locations, and suicidal tendencies respectively. We conducted data analysis using Stata version 17.0, with statistical significance determined at the following levels: p<0.1, p<0.05, and p<0.01. The detailed analytical procedures are as follows: initially, we assessed the relationship between the total number of days exposed to SHS per week each suicidal behavior among adolescents. In addition, we analyzed the associations between the number of days of SHS exposure at different locations (e.g. home, school, public places) and suicidal behaviors.

Subsequently, we examined the relationship between the frequency of SHS exposure across these locations and suicidal behaviors. Further subgroup analyses were performed among adolescents from multicultural families to explore whether these associations differed within this population. To assess whether the strength of these associations differed between multicultural and non-multicultural families, we conducted subgroup logistic regressions for each family type. We then applied the seemingly unrelated regression (SUR) model to formally compare the regression coefficients across groups. Covariates included as potential confounders in all regression models were selected based on prior literature and theoretical considerations, encompassing personal characteristics (gender, academic achievement, smoking status, grade level, living arrangement), family background (subjective family socio-economic status, father’s education level, mother’s education level), and school environment (school type, educational stage). To account for temporal and regional variations, the models also included fixed effects for survey year and city. These fixed effects control for unobserved factors that are constant within each year or city but may vary across years and locations. Finally, mediation analysis was conducted to assess the role of perceived stress in the relationship between SHS exposure and suicidal tendencies, using the Karlson–Holm–Breen (KHB) method.

## RESULTS

### Descriptive analysis results

**Figure 2:**
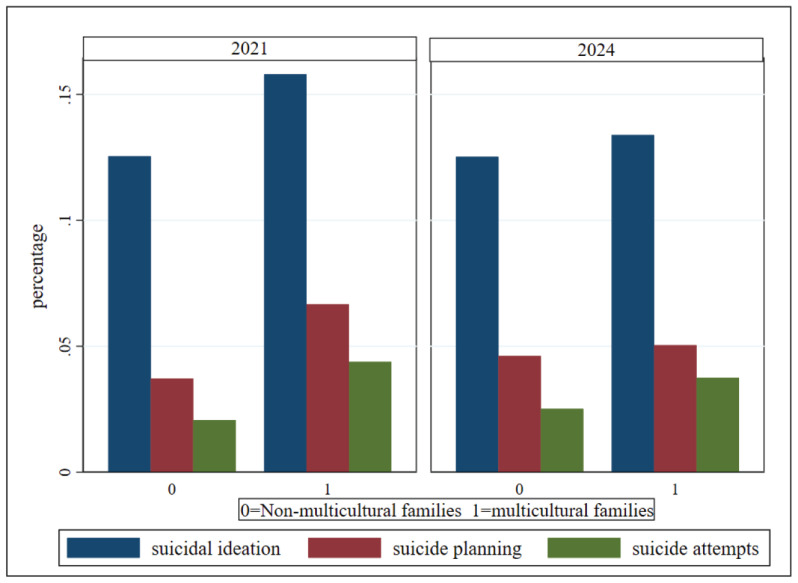
Proportion differences in suicidality between multicultural and non-multicultural families, 2021–2024 (N=84644)

Table 1 presents the descriptive statistics of the main variables. Regarding suicidality, the proportions of adolescents reporting suicidal ideation, suicide planning, and suicide attempts in the overall sample were 12.59%, 4.25%, and 2.37%, respectively, showing a decreasing trend as the severity of the behavior increased. A similar pattern was observed among adolescents from both multicultural and nonmulticultural families. Among multicultural families, the prevalence of suicidal ideation, suicide planning, and suicide attempts was 14.31%, 5.66%, and 3.99%, respectively – all significantly higher than those in non-multicultural families. This suggests a relatively stronger association with mental health difficulties among adolescents from multicultural backgrounds. Figure 2 also reveals a similar trend. SHS exposure was measured by the number of exposure days (0–7) across three settings: home, school, and public places. The average exposure days were 0.72, 0.19, and 1.11, respectively, showing an exposure intensity gradient of: public places > home > school. Adolescents from multicultural families reported significantly more days of SHS exposure at home compared to those from non-multicultural families. In the overall sample, males accounted for 48.93% and females for 51.07%, indicating a nearly balanced gender ratio. The proportion of males was slightly higher in multicultural families. Middle school students accounted for 56.05% of the total sample, while high school students made up 43.95%. The mean academic performance score was 3.14, indicating an upper middle level overall. However, adolescents from multicultural families showed significantly poorer academic performance, with higher proportions rated as ‘poor’ or ‘below average’ and lower proportions rated as ‘good’ or ‘excellent,’ compared to those from non-multicultural families. Most students attended co-educational schools. In terms of living arrangements, over 90% of adolescents lived with their families, although the proportion was slightly lower among those from multicultural families. The average perceived family economic status was 3.42, reflecting a moderate-income level, but there were significant differences between groups. Overall, 60.0% of fathers had a college education. In contrast, only 24.0% of fathers in multicultural families held a college degree, significantly lower than the 61.0% observed in non-multicultural families. Moreover, the proportion of fathers with education at the junior high school level or below (39.0% vs 20.0%), as well as those classified in the ‘Other’ category, was significantly higher among multicultural families. A comparable pattern was observed in mothers’ education level.

**Table 1:** Descriptive statistics for all variables, South Korea, 2021–2024

*Variables*	*Overall* *(N=84644)*		*Non-multicultural families* *(N=81764)*		*Multicultural families* *(N=2880)*	
	*%*	*n*	*%*	*n*	*%*	*n*
**Suicidal ideation**, mean	0.13		0.13		0.14	
No	87.41	73984	87.47	71516	85.69	2468
Yes	12.59	10660	12.53	10248	14.31	412
**Suicide planning,** mean	0.04		0.04		0.06	
No	95.75	81044	95.80	78327	94.34	2717
Yes	4.25	3600	4.20	3437	5.66	163
**Suicide attempts,** mean	0.02		0.02		0.04	
No	97.63	82639	97.69	79874	96.01	2765
Yes	2.37	2005	2.31	1890	3.99	115
**SHS exposure at home** (0–7), mean	0.72		0.71		1.01	
**SHS exposure at school** (0–7), mean	0.19		0.19		0.18	
**SHS exposure in public places** (0–7), mean	1.11		1.12		0.97	
**Gender,** mean	0.49		0.49		0.49	
Male	48.93	41418	48.93	40007	51.01	1469
Female	51.07	43226	51.07	41757	48.99	1411
**Academic achievement (1–5),** mean	3.14		3.15		1.67	
Poor	0.09	7309	0.08	6939	0.13	370
Below average	0.22	18653	0.22	17795	0.30	858
Average	0.30	25233	0.30	24366	0.30	867
Good	0.26	22173	0.26	21592	0.20	581
Excellent	0.13	11276	0.14	11072	0.07	204
**Smoked,** mean	0.08		0.08		0.09	
No	92.32	78146	92.36	75521	91.15	2625
Yes	7.68	6498	7.64	6243	8.85	255
**Grade** (1–6), mean	3.27		3.28		2.88	
1st junior high school	0.20	16665	0.19	15905	0.26	760
2nd junior high school	0.19	15996	0.19	15386	0.21	610
3rd junior high school	0.17	14784	0.17	14237	0.19	547
1st high school	0.16	13379	0.16	12988	0.14	391
2nd high school	0.15	12697	0.15	12373	0.11	324
3rd high school	0.13	11123	0.13	10875	0.09	248
**School type,** mean	1.99		1.98			
Girls’ school	0.17	14288	0.17	13824	0.16	464
Co-educational	0.68	57540	0.68	55478	0.72	2062
Boys’ school	0.15	12816	0.15	12462	0.12	354
**Education level,** mean	0.44		0.44		0.33	
Middle school	56.05	47445	55.68	45528	66.56	1917
High school	43.95	37199	44.32	36236	33.44	963
**Residence,** mean	1.11		1.10		1.18	
With family members	0.96	81420	0.96	78730	0.93	2690
At relatives’ homes	0.00	294	0.00	269	0.01	25
Boarding or renting independently	0.00	284	0.00	255	0.01	29
In dormitory	0.03	2524	0.03	2408	0.04	116
In children’s welfare institution	0.00	122	0.00	102	0.01	20
**Family economic situation** (1–5), mean	3.42		3.44		3.04	
Disadvantaged	0.01	1217	0.01	1109	0.04	108
Low income	0.08	7163	0.08	6636	0.18	527
Middle income	0.48	40295	0.47	38735	0.54	1560
High income	0.31	26375	0.32	25857	0.18	518
Affluent	0.11	9594	0.12	9427	0.06	167
**Father’s education level,** mean	2.97		2.98		2.89	
Junior middle school	0.01	1010	0.01	746	0.09	264
Senior middle school	0.20	16606	0.19	15750	0.30	856
University	0.60	50670	0.61	49980	0.24	690
Other	0.19	16358	0.19	15288	0.37	1070
**Mother’s education level,** mean	2.94		2.94		3.04	
Junior middle school	0.01	817	0.01	556	0.09	261
Senior middle school	0.22	18554	0.22	17897	0.23	657
University	0.59	50202	0.61	49524	0.24	678
Other	0.18	15071	0.17	13787	0.45	1284

**Figure 3:**
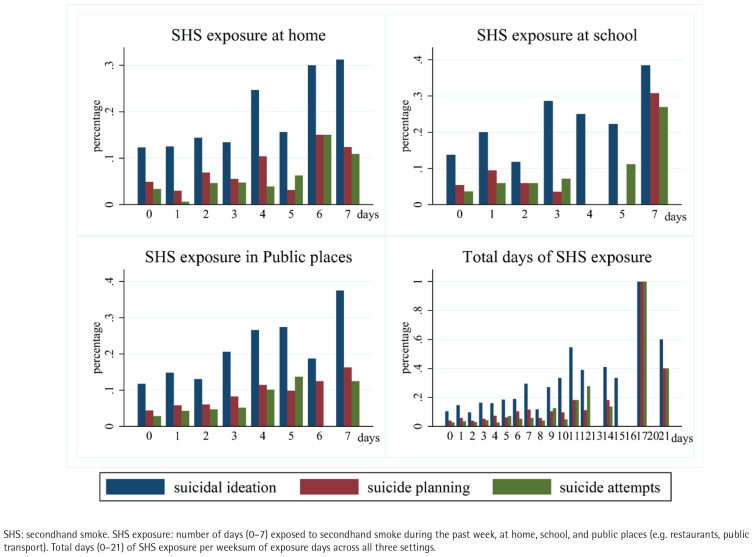
SHS exposure and suicide in different places, where the x-axis indicates the total days of SHS exposure and the y-axis shows the corresponding proportion of suicidality, 2021–2024 (N=2800)

In Figure 3, the proportion of students experiencing suicidal ideation rises steadily with increased days of SHS exposure, indicating a potential association between frequent exposure and heightened suicidality among adolescents (‘What is the impact of environmental tobacco smoke exposure on suicidality among multicultural youth? What potential mechanisms underlie this relationship?’).

### Regression results

Table 2 presents the association between the number of days adolescents are exposed to SHS and their suicidal tendencies. The findings indicate a significant positive association between the number of days of SHS exposure and adolescents’ suicidal ideation, suicide plans, and suicide attempts, after controlling for other factors. Among adolescents from non-multicultural families, each additional total day of SHS exposure per week is associated with an 8.0% increase in the likelihood of suicidal ideation (AOR=1.080; 95% CI: 1.073–1.086). The likelihood of suicide planning and suicide attempts increase by 8.6% (AOR=1.086; 95% CI: 1.076–1.096) and 8.2% (AOR=1.082; 95% CI: 1.069–1.095), respectively. Among multicultural families, the association is even more pronounced. SHS exposure is significantly associated with suicidal ideation (AOR=1.117; 95% CI: 1.084–1.151), suicide planning (AOR=1.095; 95% CI: 1.052–1.139), and suicide attempts (AOR=1.117; 95% CI: 1.069–1.168). Crude odds ratios (OR) and 95% confidence intervals (CI) are presented in Supplementary file Table 1.

**Table 2:** Logit regression results on the relationship between the total days of secondhand smoke exposure among adolescents and suicide, South Korea, 2021–2024 (N=84644)

	*Suicidal ideation* *AOR (95%CI)*		*Suicide planning* *AOR (95%CI)*		*Suicide attempts* *AOR (95%CI)*	
	*Non-multicultural* *families*	*Multicultural families*	*Non-multicultural* *families*	*Multicultural families*	*Non-multicultural* *families*	*Multicultural families*
**Total days of secondhand smoke exposure per week**	1.080*** (1.073–1.086)	1.117*** (1.084–1.151)	1.086*** (1.076–1.096)	1.095*** (1.052–1.139)	1.082*** (1.069–1.095)	1.117*** (1.069–1.168)
**Gender**	0.521*** (0.494–0.549)	0.642*** (0.493–0.835)	0.562*** (0.515–0.612)	0.654** (0.443–0.966)	0.485*** (0.432–0.545)	0.866 (0.546–1.374)
**Academic achievement**	0.914*** (0.896–0.932)	0.898** (0.810–0.994)	0.922*** (0.893–0.951)	0.982 (0.844–1.142)	0.831*** (0.797–0.867)	0.742*** (0.616–0.893)
**Smoked**	2.123*** (1.980–2.276)	1.983*** (1.413–2.783)	2.438*** (2.198–2.706)	2.545*** (1.614–4.013)	2.982*** (2.621–3.392)	3.522*** (2.160–5.743)
**Grade**	0.997 (0.971–1.023)	0.952 (0.831–1.092)	0.929*** (0.890–0.970)	0.841* (0.685–1.033)	0.894*** (0.844–0.947)	0.738** (0.576–0.946)
**School type**	0.995 (0.950–1.042)	0.981 (0.769–1.252)	0.985 (0.913–1.061)	1.005 (0.694–1.455)	1.062 (0.961–1.175)	0.921 (0.590–1.437)
**Education level**	0.712*** (0.652–0.778)	0.745 (0.467–1.187)	0.790*** (0.683–0.913)	1.060 (0.524–2.144)	0.803** (0.662–0.975)	1.656 (0.719–3.810)
**Residence**	1.145*** (1.102–1.189)	1.162* (0.999–1.353)	1.193*** (1.125–1.265)	1.123 (0.909–1.388)	1.218*** (1.130–1.314)	1.150 (0.904–1.463)
**Family economic situation**	0.839*** (0.817–0.861)	0.712*** (0.622–0.814)	0.842*** (0.807–0.878)	0.692*** (0.569–0.842)	0.840*** (0.795–0.888)	0.817* (0.651–1.026)
**Father’s education level**	0.951** (0.914–0.989)	0.961 (0.848–1.089)	0.961 (0.902–1.024)	0.848* (0.706–1.019)	0.864*** (0.795–0.939)	0.866 (0.694–1.081)
**Mother’s education level**	0.995 (0.956–1.036)	0.940 (0.831–1.063)	0.991 (0.928–1.057)	1.028 (0.856–1.234)	1.036 (0.951–1.129)	0.952 (0.765–1.184)
**Empirical p-value**	0.019**		0.289		0.046**	
**Year FE**	Yes	Yes	Yes	Yes	Yes	Yes
**City FE**	Yes	Yes	Yes	Yes	Yes	Yes
**N**	81764	2880	81764	2880	81764	2880
**pseudo R^2^**	0.0450	0.0877	0.0435	0.0894	0.0553	0.1226

AOR: adjusted odds ratio. FE: fixed effects.

* p<0.1.

** p<0.05.

*** p<0.01.

Several covariates were significant. In multicultural families, female adolescents had higher odds of suicidal ideation (AOR=0.642; 95% CI: 0.493–0.835) and suicide planning (AOR=0.654; 95% CI: 0.443–0.966) compared to males. Higher academic achievement was associated with lower odds of suicidal ideation (AOR=0.898; 95% CI: 0.810–0.994) and suicide attempts (AOR=0.742; 95% CI: 0.616–0.893). Smoking experience increased the likelihood of suicidal ideation (AOR=1.983; 95% CI: 1.413–2.783), suicide planning (AOR=2.545; 95% CI: 1.614–4.013), and suicide attempts (AOR=3.522; 95% CI: 2.160–5.743). Advancing grade level was associated with lower odds of suicide attempts (AOR=0.738; 95% CI: 0.576–0.946). Adolescents from economically advantaged families in multicultural settings exhibited lower suicidal tendencies. Specifically, these adolescents had reduced likelihood of suicidal ideation (AOR=0.712; 95% CI: 0.622–0.814), suicide planning (AOR=0.692; 95% CI: 0.569–0.842), and suicide attempts (AOR=0.817; 95% CI: 0.651–1.026) compared to peers from less affluent families. Furthermore, living arrangements, educational stage, and father’s education level showed significant effects in some models. However, mother’s education level did not show a significant association with adolescents’ suicidal tendencies.

Regression analyses indicated that a greater number of days of SHS exposure was significantly associated with increased odds of suicide-related behaviors among adolescents in both multicultural and non-multicultural families. The associations for suicidal ideation and suicide attempts were stronger among multicultural families. These findings were obtained using SUR model.

The model estimated results separately for multicultural and non-multicultural families based on SHS exposure scenarios. Table 3 presents logit regression results examining the relationship between the SHS exposure location diversity and adolescents’ suicide-related behaviors. Controlling for other factors, the frequency of SHS exposure at different locations is significantly positively associated with adolescents’ suicidal ideation, suicide plans, and suicide attempts. In non-multicultural families, each one-unit increase in exposure frequency corresponds to increased likelihood of suicidal ideation (AOR=1.352; 95% CI: 1.316–1.388), suicide planning (AOR=1.397; 95% CI: 1.340–1.457), and suicide attempts (AOR=1.407; 95% CI: 1.331–1.487). Crude odds ratios (OR) and 95% confidence intervals (CI) are presented in Supplementary file Table 2.

**Table 3:** Logit regression results on the relationship between secondhand smoke exposure location frequencies and suicide, South Korea, 2021–2024 (N=84644)

	*Suicidal ideation* *AOR (95%CI)*		*Suicide planning* *AOR (95%CI)*		*Suicide attempts* *AOR (95%CI)*	
	*Non-multicultural* *families*	*Multicultural* *families*	*Non-multicultural* *families*	*Multicultural* *families*	*Non-multicultural* *families*	*Multicultural* *families*
**SHS exposure locations and frequencies**	1.352***	1.422***	1.397***	1.395***	1.407***	1.524***
	(1.316–1.388)	(1.247–1.621)	(1.340–1.457)	(1.153–1.689)	(1.331–1.487)	(1.222–1.902)
**Gender**	0.520***	0.636***	0.561***	0.646**	0.486***	0.846
	(0.493–0.548)	(0.489–0.826)	(0.514–0.611)	(0.438–0.952)	(0.433–0.546)	(0.535–1.337)
**Academic achievement**	0.910***	0.898**	0.917***	0.984	0.827***	0.748***
	(0.893–0.928)	(0.811–0.994)	(0.888–0.946)	(0.846–1.144)	(0.792–0.862)	(0.621–0.900)
**Smoked**	2.198***	2.246***	2.547***	2.887***	3.091***	4.094***
	(2.051–2.355)	(1.616–3.121)	(2.298–2.823)	(1.859–4.484)	(2.721–3.512)	(2.555–6.561)
**Grade**	1.001	0.954	0.933***	0.842*	0.897***	0.738**
	(0.975–1.027)	(0.833–1.093)	(0.894–0.974)	(0.686–1.033)	(0.847–0.950)	(0.576–0.944)
**School type**	0.992	0.969	0.981	1.007	1.058	0.916
	(0.948–1.039)	(0.761–1.235)	(0.910–1.058)	(0.696–1.458)	(0.957–1.170)	(0.588–1.429)
**Education level**	0.712***	0.735	0.789***	1.046	0.805**	1.622
	(0.652–0.778)	(0.463–1.168)	(0.683–0.912)	(0.518–2.111)	(0.663–0.976)	(0.708–3.717)
**Residence**	1.131***	1.157*	1.175***	1.133	1.201***	1.163
	(1.089–1.175)	(0.997–1.343)	(1.108–1.246)	(0.920–1.394)	(1.114–1.295)	(0.921–1.470)
**Family economic situation**	0.837***	0.702***	0.840***	0.681***	0.840***	0.798*
	(0.815–0.859)	(0.614–0.803)	(0.805–0.876)	(0.560–0.828)	(0.794–0.888)	(0.634–1.003)
**Father’s education level**	0.948***	0.956	0.959	0.847*	0.864***	0.863
	(0.912–0.987)	(0.844–1.082)	(0.900–1.022)	(0.706–1.017)	(0.795–0.938)	(0.693–1.075)
**Mother’s education level**	0.991	0.929	0.984	1.011	1.029	0.935
	(0.952–1.031)	(0.822–1.049)	(0.922–1.050)	(0.844–1.213)	(0.945–1.121)	(0.753–1.161)
**Empirical p-value**	0.408		0.811		0.361	
**Year FE**	Yes	Yes	Yes	Yes	Yes	Yes
**City FE**	Yes	Yes	Yes	Yes	Yes	Yes
**N**	81764	2880	81764	2880	81764	2880
**pseudo R^2^**	0.0438	0.0778	0.0419	0.0836	0.0548	0.1138

AOR: adjusted odds ratio. FE: fixed effects.

* p<0.1.

** p<0.05.

*** p<0.01.

In multicultural families, each one-unit increase in the frequency of adolescents’ SHS exposure across various locations significantly raises the likelihood of suicidal ideation (AOR=1.422; 95% CI: 1.247–1.621), suicide planning (AOR=1.395; 95% CI: 1.153–1.689), and suicide attempts (AOR=1.524; 95% CI: 1.222–1.902). A significant positive association was found between the frequency of SHS exposure across different locations and suicidal behaviors among adolescents from multicultural families. Table 4 presents a detailed analysis of the association between SHS exposure at different locations and suicide-related behaviors among multicultural adolescents. Logit regression models were conducted separately for exposure in the home, school, and public places, controlling for year and city fixed effects. Specifically, adolescents exposed to SHS more frequently at home had higher odds of suicidal ideation (AOR=1.143; 95% CI: 1.091–1.196), suicide planning (AOR=1.110; 95% CI: 1.039–1.187), and suicide attempts (AOR=1.152; 95% CI: 1.069–1.242).

**Table 4:** Logit regression results on the relationship between secondhand smoke exposure locations and suicide among multicultural youth, South Korea, 2021–2024 (N=2880)

	*Home* *AOR (95% CI)*	*School* *AOR (95% CI)*	*Public places* *AOR (95% CI)*
**Suicidal ideation**	1.143*** (1.091–1.196)	1.163*** (1.044–1.296)	1.190*** (1.123–1.261)
Year FE	Yes	Yes	Yes
City FE	Yes	Yes	Yes
pseudo R^2^	0.0790	0.0693	0.0802
**Suicide planning**	1.110*** (1.039–1.187)	1.170** (1.026–1.333)	1.177*** (1.086–1.276)
Year FE	Yes	Yes	Yes
City FE	Yes	Yes	Yes
pseudo R^2^	0.0817	0.0785	0.0860
**Suicide attempts**	1.152*** (1.069–1.242)	1.229*** (1.068–1.413)	1.199*** (1.094–1.315)
Year FE	Yes	Yes	Yes
City FE	Yes	Yes	Yes
pseudo R^2^	0.1127	0.1073	0.1139

AOR: adjusted odds ratio. FE: fixed effects.

* p<0.1.

** p<0.05.

*** p<0.01.

Exposure to SHS in schools significantly increases the likelihood of suicidal ideation (AOR=1.163; 95% CI: 1.044–1.296), suicide planning (AOR=1.170; 95% CI: 1.026–1.333), and suicide attempts (AOR=1.229; 95% CI: 1.068–1.413) among adolescents, with all associations reaching statistical significance. However, secondhand smoke not only threatens physical health but also appears to harm psychological well-being, potentially increasing the likelihood of suicidal thoughts and behaviors.

Exposure to SHS in public places is significantly associated with all three suicide-related behaviors at p<0.01. Multicultural adolescents exposed to SHS in these settings were more likely to report suicidal ideation (AOR=1.190; 95% CI: 1.123–1.261), develop suicide plans (AOR=1.177; 95% CI: 1.086–1.276), and engage in suicide attempts (AOR=1.199; 95% CI: 1.094–1.315).

### The analysis of mediation effect

The progression from suicidal ideation to planning and ultimately attempting suicide represents an escalating level of risk among adolescents. This study focuses solely on the mediating effect of perceived stress in the association between the number of days of SHS exposure and suicide attempts. Table 5 presents perceived stress as a mediating variable. The results (Model 1) show that each additional total day of SHS exposure per week increases the likelihood of adolescents attempting suicide (AOR=1.117; 95% CI: 1.069–1.168). Model 2 reveals a significant positive association between SHS exposure and adolescents’ perceived stress (AOR=1.085; 95% CI: 1.060–1.110). Model 3 indicates a strong positive association between perceived stress and suicide attempts (AOR=2.110; 95% CI: 1.660–2.681). Furthermore, after controlling for perceived stress, the effect of SHS exposure on suicide attempts diminishes, suggesting that perceived stress partially mediates this relationship. These findings highlight the importance of addressing both environmental risk factors and psychological stress when developing interventions to prevent suicide attempts among adolescents.

**Table 5:** Mediating effect of perceived stress, South Korea, 2021–2024 (N=2880)

	*Model 1*	*Model 2*	*Model 3*
	*Suicide attempts* *AOR (95% CI)*	*Perceived stress* *AOR (95% CI)*	*Suicide attempts* *AOR (95% CI)*
Total days of SHS exposure per week	1.117*** (1.069–1.168)	1.085*** (1.060–1.110)	1.095*** (1.044–1.147)
Perceived stress			2.110*** (1.660–2.681)
Year FE	Yes	Yes	Yes
City FE	Yes	Yes	Yes
pseudo R^2^	0.1226	0.0300	0.1646

AOR: adjusted odds ratio. FE: fixed effects.

* p<0.1.

** p<0.05.

*** p<0.01.

Table 6, using the KHB method for mediating effect decomposition, further demonstrates that perceived stress plays a significant mediating role in the association between SHS exposure and suicide attempts. The total effect is 0.118; the direct effect is 0.090; and the mediating effect is 0.027, accounting for 23.19% of the total effect. This indicates that 23.19% of the effect of SHS exposure on suicide attempts is indirectly mediated through perceived stress. Additionally, this study analyzed suicidal ideation and suicide planning, with perceived stress mediating 30.67% and 34.18% of the effects, respectively.

**Table 6:** KHB test for mediating effect, South Korea (2021–2024) (N=2880)

	*Suicidal ideation*	*Suicide planning*	*Suicide attempts*
Reduced	0.128	0.097	0.118
Full	0.089	0.064	0.090
Diff	0.039	0.033	0.027
Percentage of mediating effect	30.67	34.18	23.19

Reduced: total effect. Full: direct effect. Diff: mediating effect. KHB: Karlson-Holm-Breen method.

This indirect pathway through perceived stress contributes significantly to the overall association between SHS exposure and suicidality. These findings underscore the need for comprehensive strategies addressing both the direct and indirect pathways through which SHS exposure is associated with the likelihood of suicidal behavior in adolescents.

## DISCUSSION

The study reveals four key findings that enhance comprehension of the interplay between environmental health hazards and psychosocial stressors in this demographic. These results align with prior research suggesting that SHS exposure is connected to heightened mental health risks^17,18^. Various mechanisms could elucidate this relationship. SHS exposure may function not only as a biological risk factor but also as an indicator of family dysfunction, chronic stress, or inadequate parental supervision, all established risk factors for adolescent suicidality^22^. From a neurobiological standpoint, sustained exposure to nicotine, even passively, has been associated with changes in neurotransmitter systems like serotonin and dopamine, which play roles in mood regulation and impulse control^23^. While the direction and strength of the associations were similar across family types, the magnitude of this association tended to be greater among adolescents from multicultural families^12^. This could indicate their increased susceptibility due to cumulative disadvantages such as social exclusion, economic hardship, and cultural or linguistic barriers, which can compound the psychological impact of SHS exposure^24^. In light of these findings, efforts to reduce SHS exposure – particularly within households – and to develop culturally sensitive mental health interventions could potentially contribute to lowering suicidal behaviors among at-risk adolescent populations.

The study’s second key finding underscores a significant positive association between SHS exposure frequency in various settings and suicidal ideation, planning, and attempts among multicultural and non-multicultural adolescents in South Korea. Notably, a stronger-magnitude association was observed in adolescents from multicultural backgrounds, suggesting that the diversity and ubiquity of exposure locations may serve as potential psychosocial vulnerability indicators in youth populations. These results align with prior research suggesting that SHS exposure in diverse contexts, such as at home, in public areas, or among peers, can exacerbate psychological distress. Reducing such exposure may play a crucial role in mitigating the risks of suicidal ideation, suicide planning, and suicide attempts among these adolescents. For example, Lee and Kim^25^ demonstrated a significant link between SHS exposure and heightened risks of depression and suicidal ideation in adolescents, particularly when exposure occurred in multiple settings. Frequent SHS exposure in various locations may not only signify increased physiological exposure to harmful smoke but also reflect cumulative social and emotional risk factors. Adolescents regularly exposed to SHS in multiple environments may reside in tumultuous, unsupervised, or socially disadvantaged settings, all recognized as contributors to suicidal behavior^16^.

Among multicultural adolescents, the amplified association may stem from their distinct sociocultural vulnerabilities. These individuals often confront challenges such as identity conflicts, marginalization, and limited access to social resources, intensifying the psychological impact of SHS exposure in significant social settings like home and school^26^. The observed elevated likelihood associated with multi-location SHS exposure, especially among multicultural adolescents, highlight the complex interplay between environmental and psychosocial factors influencing suicidal behaviors. These findings suggest that addressing SHS exposure alone may be insufficient without considering the broader contextual factors such as family dynamics, social support, and cultural challenges. Future research and interventions could explore integrated approaches that combine efforts to reduce SHS exposure with culturally sensitive mental health support and family engagement to better address the needs of this vulnerable population.

This third key finding underscores the substantial psychological risks linked to SHS exposure in various settings – home, school, and public spaces – among adolescents from multicultural families in South Korea. The consistent relationship between SHS exposure in each location and an increased likelihood of suicidal ideation, planning, and attempts suggests that the exposure setting plays an active role in accumulating psychosocial stress, rather than being merely a passive backdrop. These results align with prior research by Kim et al.^25^, demonstrating a strong connection between SHS exposure in multiple social environments and heightened depressive symptoms and suicidality in Korean youth. SHS not only threatens physical health but also appears to harm psychological well-being, potentially increasing the likelihood of suicidal thoughts and behaviors.

Other studies also highlight that SHS exposure, particularly within familial and educational contexts, may indicate broader issues in parental supervision, family cohesion, and peer relationships – factors known to influence adolescent mental health risks^27,28^. Various mechanisms could explain these connections. Exposure to SHS at home may mirror parental smoking habits, ineffective family communication, or conflict-ridden domestic atmospheres, all recognized as risk factors for suicidal behaviors^29^. SHS exposure at school or in public areas could also signify social neglect, normalization of peer group risks, or a general community acceptance of smoking, all of which can further isolate vulnerable adolescents^30^. These dynamics may be particularly pronounced in multicultural families, where intergenerational acculturation gaps, socio-economic challenges, and discrimination can exacerbate feelings of isolation and despair among young individuals. Given these findings, interventions should not only focus on reducing SHS exposure but also acknowledge the environmental context as indicative of deeper relational and institutional vulnerabilities^31^. Addressing smoke-free environments in households and educational settings, together with culturally sensitive mental health assessments for multicultural adolescents, represents an important consideration for understanding and potentially mitigating suicide likelihood within this demographic.

This study demonstrates that perceived stress serves as a partial mediator in the link between SHS exposure and suicidality among adolescents in multicultural families in South Korea. Perceived stress is an important mediating variable linking risk factors to suicidal behaviors. Notably, the proportion of the mediating effect of perceived stress differs across the three types of suicidalities. This variation suggests that suicide attempts are associated with both high levels of stress and SHS exposure. The mediating effect is strongest for suicide planning, indicating that perceived stress plays the most significant explanatory role in the transition from suicidal ideation to planning. This may reflect the critical role of stress in shaping concrete decision-making. In contrast, the mediating effect is weakest for suicide attempts, suggesting that additional direct drivers – such as immediate environmental stimuli or individual coping strategies – may influence the progression from ideation and planning to actual attempts. One plausible explanation is rooted in the threshold model of suicidal behavior, which suggests that while ideation may stem from persistent psychosocial challenges, the progression to an attempt typically necessitates acute psychological distress or emotional instability^32^. SHS exposure, indicative of adverse environments, could elevate overall stress levels, yet it is only under specific circumstances – such as prolonged exposure or inadequate coping mechanisms – that this stress leads to action^25^. This dynamic is particularly pertinent for multicultural adolescents who often confront compounded stressors related to discrimination, familial discord, and cultural alienation^33^. While these stressors may not directly trigger suicidal ideation, they can exacerbate emotional crises, thereby heightening the likelihood of suicide attempts when combined with SHS exposure. From a public health perspective, these findings highlight the importance of further research to explore the complex relationships between SHS exposure, perceived stress, and suicidality among multicultural adolescents. Given the cross-sectional design of this study, causal relationships cannot be confirmed. Therefore, longitudinal or cohort studies are needed to clarify temporal sequences and underlying mechanisms. Future research could also investigate how emotional resilience and cultural factors influence these associations in multicultural youth, ultimately informing tailored prevention strategies.

### Limitations

Firstly, the cross-sectional design hinders causal inference and may be subject to potential reverse causality, necessitating longitudinal data to elucidate the temporal dynamics among SHS exposure, perceived stress, and suicidality effectively. Additionally, the KHB method, while useful for assessing mediation, is a statistical approach and cannot definitively establish causal relationships. Secondly, the use of self-reported measures for SHS exposure, stress, and suicidal behaviors may introduce recall bias, social desirability effects, and information bias caused by potential misclassification, which could affect the accuracy of the results. Thirdly, despite focusing on multicultural adolescents, the study does not fully consider the heterogeneity within this population, such as variations in immigration status, parental nationality, or levels of acculturation, which could moderate the observed associations. Fourthly, unaccounted confounding variables – such as prior mental health history, access to psychological counseling, and family socio-economic status – represent residual confounding that was not thoroughly controlled for and may have influenced the observed associations. Lastly, the findings are based on data from the KYRBS, conducted among South Korean adolescents, which may limit generalizability to other countries or settings with different cultural, social, or environmental contexts.

## CONCLUSIONS

This study reveals a significant association between frequent exposure to SHS and an increased likelihood of suicidal ideation, suicide planning, and suicide attempts among multicultural adolescents in South Korea. The cumulative frequency of SHS exposure in various settings – such as home, school, and public places – further increases the likelihood of suicidality, particularly impacting this vulnerable population. These findings suggest that pervasive environmental exposure to SHS may function as a psychosocial vulnerability indicator. Specifically, SHS exposure in specific contexts, notably at home and in schools, independently correlates with heightened likelihood of suicidal behavior, potentially amplifying familial and institutional stressors that contribute to mental health challenges. Additionally, perceived stress serves as a partial mediator in the relationship between SHS exposure and suicide attempts, emphasizing the role of stress in the progression from suicidal ideation to actions among multicultural adolescents.

## Supplementary Material



## Data Availability

The data supporting this research are available from the following source: https://www.kdca.go.kr
