# Supplementary material for: A cross-sectional study on the association between secondhand smoke exposure and suicide among adolescents in multicultural families: The mediating role of perceived stress
Source: Tob Induc Dis. 2025 Oct 21;23:10.18332/tid/209551. doi: 10.18332/tid/209551 (PMC12535225; doi:10.18332/tid/209551)
Supplement: Supplementary file 1 [file TID-23-160-s1.pdf]

Supplementary Table 1. Crude Logit Regression Results on the Relationship between the Total Days of Secondhand Smoke Exposure among Adolescents and Suicide, South Korea (2021, 2024) (N =84644)

	Suicidal ideation OR(95%CI)			Suicide planning OR(95%CI)			Suicide attempts OR(95%CI)		
	Overall	Non -multicultural families	Multicultural families	Overall	Non -multicultural families	Multicultural families	Overall	Non -multicultural families	Multicultural families
Total days of SHS exposure	1.102***	1.101***	1.143***	1.109***	1.108***	1.130***	1.115***	1.111***	1.160***
	[1.096,1.109]	[1.094,1.107]	[1.111,1.176]	[1.100,1.118]	[1.098,1.117]	[1.088,1.172]	[1.103,1.127]	[1.099,1.124]	[1.114,1.208]
Year FE	Yes	Yes	Yes	Yes	Yes	Yes	Yes	Yes	Yes
City FE	Yes	Yes	Yes	Yes	Yes	Yes	Yes	Yes	Yes
N	84644	81764	2880	84644	81764	2880	84644	81764	2880
pseudo R^2	0.0178	0.0172	0.0557	0.0192	0.0189	0.0565	0.0198	0.0185	0.0742

Exponentiated coefficients: OR (Odds ratio); 95% confidence intervals in brackets
* $p < 0.1$, ** $p < 0.05$, *** $p < 0.01$

Supplementary Table 2. Crude Logit Regression Results on the Relationship between Secondhand Smoke Exposure Location Frequencies and Suicide, South Korea (2021, 2024) (N =84644)

	Suicidal ideation OR(95%CI)			Suicide planning OR(95%CI)			Suicide attempts OR(95%CI)		
	Overall	Non -multicultural families	Multicultural families	Overall	Non -multicultural families	Multicultural families	Overall	Non -multicultural families	Multicultural families
SHS exposure locations and frequencies	1.458***	1.454***	1.553***	1.513***	1.511***	1.553***	1.576***	1.563***	1.734***
	[1.422,1.494]	[1.417,1.492]	[1.369,1.761]	[1.454,1.575]	[1.451,1.574]	[1.291,1.868]	[1.496,1.660]	[1.482,1.650]	[1.400,2.147]
Year FE	Yes	Yes	Yes	Yes	Yes	Yes	Yes	Yes	Yes
City FE	Yes	Yes	Yes	Yes	Yes	Yes	Yes	Yes	Yes
N	84644	81764	2880	84644	81764	2880	84644	81764	2880
pseudo R^2	0.0150	0.0148	0.0404	0.0162	0.0162	0.0447	0.0173	0.0167	0.0549

Exponentiated coefficients: OR (Odds ratio); 95% confidence intervals in brackets
* $p < 0.1$, ** $p < 0.05$, *** $p < 0.01$
Year FE: Year Fixed Effects
City FE: City Fixed Effects

© 2025 Du W. et al.
